# Association Between Plasma Fibrinogen Concentration After Cardiopulmonary Bypass and Postoperative Blood Loss in Children Undergoing Cardiac Surgery: A Retrospective Cohort Study

**DOI:** 10.7759/cureus.38245

**Published:** 2023-04-28

**Authors:** Keisuke Nishida, Taiki Kojima, Matthew P Monteleone, Fumio Watanabe

**Affiliations:** 1 Department of Anesthesiology, Aichi Children’s Health and Medical Center, Obu, JPN; 2 Division of Comprehensive Pediatric Medicine, Nagoya University Graduate School of Medicine, Nagoya, JPN; 3 Division of Cardiac Anesthesia, Cincinnati Children’s Hospital Medical Center, Ohio, USA

**Keywords:** cyanotic heart disease, postoperative blood loss, fibrinogen concentration, coagulopathy, cardiac surgery, pediatric anesthesia

## Abstract

Background

Intraoperative hypofibrinogenemia is a major factor associated with increased postoperative blood loss in adult cardiac surgery. However, previous pediatric studies on this topic did not sufficiently adjust for potential confounders and variations in surgeons’ techniques. Therefore, evidence for the association between hypofibrinogenemia and postoperative blood loss after cardiac surgery in children remains insufficient. In this study, we aimed to evaluate the association between postoperative blood loss and hypofibrinogenemia by adjusting for potential confounders and the effects of differences in surgeons’ techniques.

Methodology

This single-center, retrospective, cohort study included children who underwent cardiac surgery with cardiopulmonary bypass from April 2019 to March 2022. Multilevel logistic regression models with mixed effects were used to evaluate the association of major blood loss in the first six hours postoperatively with fibrinogen concentration at the end of cardiopulmonary bypass. The difference in the surgeon’s techniques was adjusted as a random effect for the model. The model included potential confounders identified as risk factors in previous studies.

Results

A total of 401 patients were included. A fibrinogen concentration ≤150 mg/dL (adjusted odds ratio (aOR) = 2.08; 95% confidence interval (CI) = 1.18-3.67; p = 0.011) and the presence of cyanotic disease (aOR = 2.34; 95% CI = 1.10-4.97; p = 0.027) were associated with major blood loss in the first six postoperative hours.

Conclusions

A fibrinogen concentration ≤150 mg/dL and the presence of cyanotic disease were associated with postoperative blood loss in pediatric cardiac surgery. Maintaining a fibrinogen concentration >150 mg/dL is recommended, especially for patients with cyanotic diseases.

## Introduction

Pediatric patients undergoing cardiac surgery may develop coagulopathy after cardiopulmonary bypass (CPB) due to hemodilution and consumption of coagulation factors during CPB [1]. Previous studies have shown that hypofibrinogenemia increases postoperative blood loss and the need for transfusion of blood products in pediatric cardiac surgery [2,3]. Therefore, fibrinogen supplementation may be needed to reduce blood loss after pediatric cardiac surgery when hypofibrinogenemia occurs during CPB.

In previous studies, several risk factors that increase postoperative blood loss after pediatric cardiac surgery were described, including age less than one year, the presence of cyanotic heart disease, re-sternotomy, deep hypothermia during CPB, long CPB duration, and operative complexity (Risk Adjustment for Congenital Heart Surgery (RACHS)-1 score) [4-7]. However, previous studies did not sufficiently adjust for potential confounders to explore the association between hypofibrinogenemia and postoperative blood loss. In addition, these studies did not adjust the findings for variations in surgeons’ techniques.

This retrospective cohort study aimed to evaluate the association between hypofibrinogenemia and an increase in postoperative blood loss in children undergoing cardiac surgery. We hypothesized that hypofibrinogenemia after CPB was associated with an increase in postoperative blood loss after adjusting for potential confounders and variations in surgeons’ techniques.

## Materials and methods

Study design, setting, and ethical approval

This retrospective cohort study was conducted from April 2019 to March 2022 at the Aichi Children’s Health and Medical Center, a 200-bed tertiary care children’s hospital in Japan.

Ethical approval was obtained from the local ethics committee of Aichi Children’s Health and Medical Center (approval number: 2022001, April 9, 2022). An opt-out procedure provided the patient and the patient’s family the opportunity to refuse participation in this study.

Inclusion and exclusion criteria

All patients aged <18 who underwent cardiac surgery with CPB at the Aichi Children’s Health and Medical Center from April 2019 to March 2022 were enrolled in this study. The exclusion criteria were as follows: (1) duplicate cases during the study period and (2) refusal to participate in this study through the opt-out procedure. To avoid duplication, only the first surgery was included for analysis if a patient underwent more than one surgery during the study period.

Data acquisition

Demographic data were extracted from the electronic medical, anesthesia, and pediatric intensive care unit (PICU) records of the Aichi Children’s Health and Medical Center. The collected data included information regarding the age, sex, body weight, diagnosis, presence of cyanotic heart disease, operative complexity (RACHS-2 score), re-sternotomy, length of surgery, length of anesthesia, CPB duration, duration of the aortic clamp, the minimum temperature during surgery, amount of perioperative transfusion (fresh frozen plasma (FFP) and platelet concentrate (PC)), and fibrinogen concentrate (60 mg/kg) administration. The assigned surgeons and the number of cases per surgeon were also recorded. The fibrinogen concentration and platelet count were routinely evaluated at the following points: after induction of general anesthesia (baseline), after protamine administration at the end of CPB, at admission to the PICU, and on postoperative day (POD) one in the PICU. The fibrinogen concentration was measured using the Clauss fibrinogen assay.

Definitions of hypofibrinogenemia and cyanotic heart disease

Patients were categorized into two groups according to the presence of hypofibrinogenemia after protamine administration at the end of CPB: group H with fibrinogen level ≤150 mg/dL, and group N with fibrinogen level >150 mg/dL. We applied a fibrinogen concentration threshold of 150 mg/dL that has been used in previous studies [3].

Cyanotic heart disease was defined as the presence of right-to-left intra or extracardiac shunts and the need for the following surgeries: modified Blalock-Taussig shunt, Norwood operation, total anomalous pulmonary venous connection repair, arterial switch operation, Glenn procedure, total cavopulmonary connection, intracardiac repair for tetralogy of Fallot, double-outlet right ventricle, and truncus artery repair.

Anesthetic management

American Society of Anesthesiologists standard anesthetic monitoring, arterial and central venous pressure monitoring, urinary output monitoring, and transesophageal echocardiography were performed during cardiac surgery. General anesthesia was induced with propofol (1-2 mg/kg), midazolam (0.1 mg/kg), fentanyl (2-3 µg/kg), and rocuronium (1 mg/kg). Endotracheal intubation was performed, and the patient was maintained on mechanical ventilation during the surgery. Sevoflurane (0.5-3%), propofol (5-12 mg/kg/hour), fentanyl (20-30 µg/kg), remifentanil (0.05-0.5 µg/kg/minute), and rocuronium (7-10 µg/kg/minute) were used to maintain general anesthesia based on the clinical judgment of the assigned anesthesiologist. Tranexamic acid (100 mg/kg) was administered during the induction of general anesthesia.

CPB management and transfusion strategy

Four types of CPB circuits were used in the study institution based on the patient’s body weight: the CPB priming volume was 350, 450, 800, and 1,000 mL for patients weighing <10 kg, 10-16 kg, 16-31 kg, and ≥31 kg, respectively. The minimum core temperature was controlled between 20°C and 35°C during CPB, in accordance with the surgical procedure. Modified ultrafiltration was performed after rewarming from CPB. Anticoagulation was managed with 300 U/kg of heparin before aortic cannulation, and an additional dose of heparin was administered to maintain an activating clotting time ≥400 seconds during CPB. Protamine (30 mg/kg) was administered after the completion of modified ultrafiltration.

The hematocrit (Hct) level after CPB was calculated preoperatively for all participants. The circuit was primed with 280 mL of red blood cells (RBCs) when the calculated Hct level was <21%. However, FFP was not used during CPB priming. During CPB, RBCs were utilized to maintain an Hct of 21%, and 120 mL or 240 mL of FFP was administered to patients weighing <20 kg or ≥20 kg, respectively. RBCs, FFP, PC, and fibrinogen concentrate were used after CPB based on the judgment of the assigned anesthesiologists.

Outcomes

The primary outcome was the occurrence of major blood loss in the first six postoperative hours (MBL-6). The chest drainage output was recorded as blood loss. MBL-6 was defined as an estimated blood volume (EBV) of more than 10% in the first six hours postoperatively. EBV was calculated as follows: 90 mL/kg for patients aged <1 month, 80 mL/kg for patients aged 1-12 months, and 70 mL/kg for patients aged ≥12 months [8].

Statistical analysis

Continuous variables were presented as mean (standard deviation) or median (interquartile range), and categorical variables were presented as number (percentage). The Kolmogorov-Smirnov test was used to evaluate normality for continuous variables. Multilevel logistic regression with a mixed-effect model yielding odds ratios (ORs) and 95% confidence intervals (CIs) was used to identify the association of MBL-6 with the presence of hypofibrinogenemia.

The model included potential confounders that were selected based on the findings of previous studies and the clinical experience of anesthesiologists among the research members before data collection. The potential confounders were age, CPB time, RACHS-2 score, preoperative fibrinogen level, fibrinogen level after CPB, platelet count after CPB, perioperative minimum body temperature, cyanosis, re-sternotomy, perioperative transfusion volume of FFP, PC, and fibrinogen concentrate [4-7]. The effect of the technique of each surgeon on the postoperative blood loss was adjusted as a random effect for the model. A complete case analysis was performed to construct logistic models for the missing data. Multicollinearity was assessed using variance inflation factors (VIF). A two-sided p-value of <0.05 was used to evaluate the null hypothesis for each analysis. Data were analyzed utilizing STATA 17.0 (StataCorp®︎, College Station, TX, USA).

## Results

**Figure 1:**
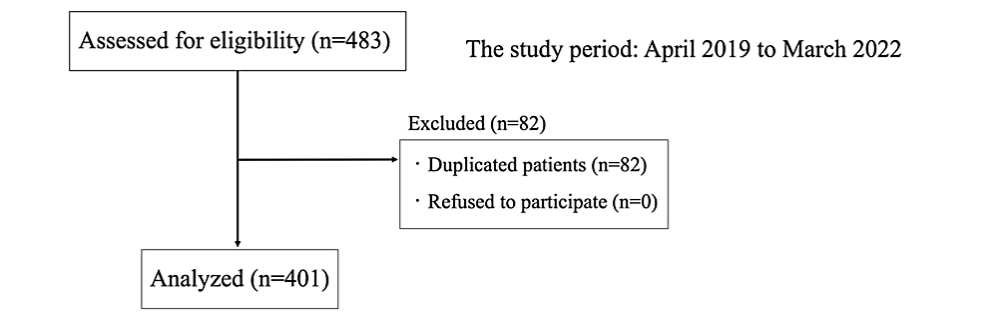
Flow diagram of the participants.

A total of 483 pediatric patients (202 in group H, 199 in group N) who underwent cardiac surgery with CPB between April 2019 and March 2022 were included in the study. The duplicate records of 82 patients were excluded, and the findings for 401 patients were analyzed (Figure 1).

The patients’ baseline and post-interventional characteristics are summarized in Table 1.

**Table 1:** Patient characteristics and postintervention parameters (n = 401). Data are presented as median (IQR) or number (proportion). ^a^: Group H, fibrinogen level ≤150 mg/dL; ^b^: Group N, fibrinogen level >150 mg/dL. IQR: interquartile range; CPB: cardiopulmonary bypass; RACHS-2: Risk Adjustment for Congenital Heart Surgery-2

	^a^Group H (n = 202)	^b^Group N (n = 199)	P-value
Patient characteristics			
Age (months), %			<0.001
<1	28 (13.9)	9 (4.5)	
1 to 12	126 (62.4)	51 (25.6)	
≥12	48 (23.8)	139 (69.9)	
Sex, male, %	114 (56.4)	101 (50.8)	0.25
Body weight (kg)(IQR)	5.8 (3.6, 7.9)	9.6 (7.3, 18.9)	<0.001
Cyanosis, %	86 (42.6)	56 (28.1)	0.003
Postintervention parameters			
RACHS-2 score, (IQR)	2 (1, 3)	1 (1, 2)	0.04
Re-sternotomy, %	72 (35.6)	55 (27.6)	0.09
Length of surgery (minutes) (IQR)	316 (264, 375)	316 (265, 385)	0.39
Length of during anesthesia (minutes) (IQR)	429 (374, 497)	428 (378, 506)	0.76
CPB duration (minutes) (IQR)	170 (130, 215)	173 (132, 227)	0.55
Aortic clamp duration (minutes) (IQR)	76 (38, 102)	82 (43, 115)	0.17
Minimum temperature (℃) (IQR)	30.3 (29.9, 31.0)	30.4 (29.9, 32.2)	0.09

Patients in group H were younger and had a lower body weight, higher proportion of cyanosis, and higher RACHS-2 scores. A total of seven surgeons were enrolled, and the numbers of cases per surgeon were 116, 129, 31, 86, 14, 24, and one, respectively. The laboratory parameters and the amount of RBC, FFP, PC, and fibrinogen concentrate administered are shown in Table 2.

**Table 2:** Perioperative transfusion of blood products and laboratory parameters. Data are presented as median (IQR), or number (proportion). ^a^: Group H, fibrinogen level ≤150 mg/dL; ^b^: Group N, fibrinogen level >150 mg/dL. *: Data on a total of 30 platelet counts after protamine administration were missing. ^†^: Data on a total of 38 fibrinogen concentration levels after protamine administration were missing. ^‡^: Data on one POD 1 fibrinogen concentration level was missing. IQR: interquartile range; FFP: fresh frozen plasma; PC: platelet concentrate; PICU: pediatric intensive care unit; POD: postoperative day

Perioperative transfusion of blood products	Group H (n = 202)	Group N (n = 199)	P-value
FFP (mL/kg)v (IQR)	6.9 (3.74, 10.7)	4.2 (0.0, 7.3)	<0.001
PC (mL/kg) (IQR)	0.0 (0.0, 6.3)	0.0 (0.0, 3.3)	<0.001
Fibrinogen concentrate, %	49 (24.3)	22 (11.1)	0.001
Laboratory parameters			
Baseline			
Platelet count (×10^4^) (IQR)	31.2 (25.5, 38.3)	27.9 (23.4, 32.4)	<0.001
Fibrinogen concentration (mg/dL) (IQR)	169 (152, 192)	219 (191, 253)	<0.001
After protamine administration			
Platelet counts (×10^4^) (IQR)^*^	10.7 (8.2, 13.8)	13.3 (9.9, 16.8)	<0.001
Fibrinogen concentration (mg/dL) (IQR)^†^	126 (109, 140)	180 (164, 210)	<0.001
Admission to the PICU			
Platelet counts (×10^4^) (IQR)	13.7 (10.5, 17.0)	15.7 (12.6, 19.4)	<0.001
Fibrinogen concentration (mg/dL) (IQR)	140 (124, 165)	181 (160, 210)	<0.001
POD 1			
Platelet counts (×10^4^) (IQR)	18.7 (15.1, 24.1)	19.5 (15.5, 23.0)	0.94
Fibrinogen concentration (mg/dL) (IQR)^‡^	252 (225, 282)	284 (254, 324)	<0.001

The amount of FFP, PC, and fibrinogen concentrate administered was larger in group H than that in group N. The platelet counts and fibrinogen concentration at the four time points, except for platelet count at POD one, were significantly lower in group H.

Fibrinogen concentration ≤150 mg/dL after protamine administration at the end of CPB was associated with MBL-6 (adjusted OR (aOR) = 2.08; 95% CI = 1.18-3.67; p = 0.011). The presence of cyanotic disease was associated with MBL-6 (aOR = 2.34; 95% CI = 1.10-4.97; p = 0.027) (Table 3).

**Table 3:** Multilevel logistic regression with mixed effects for the occurrence of major blood loss in the first six hours postoperatively. Multicollinearity was assessed by calculating the variance inflation factor (VIF) for each variable. All VIF values were <5 (maximum VIF = 4.91). Thirty patients were excluded because of missing data. aOR: adjusted odds ratio; CI: confidence interval; CPB: cardiopulmonary bypass; RACHS-2: Risk Adjustment for Congenital Heart Surgery-2; FFP: fresh frozen plasma; PC: platelet concentration

Variables	aOR	95% CI	P-value
Laboratory parameters after protamine administration			
Fibrinogen concentration (mg/dL)			
≤150	2.08	1.18–3.67	0.011
>150	1 (Reference)		
Platelet count (×10^4^)	0.95	0.89–1.01	0.10
Age (months)			
<1	0.81	0.21–3.18	0.76
1 to 12	1.40	0.68–2.86	0.36
≥12	1 (Reference)		
CPB duration (minutes)	1.00	1.00–1.01	0.10
RACHS-2 score	1.13	0.81–1.60	0.47
Minimum temperature (℃)	0.97	0.86–1.09	0.62
Cyanosis	2.34	1.10–4.97	0.027
Re-sternotomy	1.01	0.58–1.97	0.83
Perioperative transfusion of blood products			
FFP (mL/kg)	1.05	1.02–1.08	<0.001
PC (mL/kg)	0.98	0.92–1.04	0.52
Fibrinogen concentrate	0.80	0.34–1.83	0.59

## Discussion

This single-center, retrospective, cohort study investigated the association between the presence of hypofibrinogenemia after protamine administration at the end of CPB and the occurrence of MBL-6. The findings were adjusted for potential confounders based on previous studies and the clustering effect of surgeons’ techniques by applying a multilevel logistic regression analysis with mixed effects. The results showed that fibrinogen concentration ≤150 mg/dL after protamine administration was associated with the occurrence of MBL-6. Furthermore, the presence of cyanotic disease was associated with MBL-6.

Fibrinogen plays an important role in the coagulation system following protamine administration at the end of CPB. Clot formation is observed with a fibrinogen concentration of at least 75 mg/dL [9]. Critically low fibrinogen concentrations are associated with postoperative blood loss [2,3]. Hypofibrinogenemia might be observed at the end of CPB in neonates and infants because their preoperative fibrinogen level has a tendency to be lower and their lower blood volume may be associated with the bigger impact of hemodilution by CPB compared with patients aged ≥1 year. Moreover, high RACHS-2 scores may be associated with hypofibrinogenemia at the end of CPB because surgeries during the neonatal period and infancy have relatively high scores. High fibrinogen concentrations may be required to prevent postoperative blood loss, especially given the platelet dysfunction caused by CPB and hypothermia during cardiac surgery [10]. Evidence for the association of hypofibrinogenemia with postoperative blood loss in pediatric patients undergoing cardiac surgery with CPB is limited. A previous study showed that post-CPB fibrinogen concentration ≤150 mg/dL was associated with postoperative blood loss [2]. The guidelines proposed by the Network for the Advancement of Patient Blood Management, Hemostasis and Thrombosis (NATA) recommend that patients with fibrinogen concentration ≤150 mg/dL should be treated with cryoprecipitate or fibrinogen concentrate [11]. Therefore, we investigated the association between fibrinogen concentration ≤150 mg/dL and postoperative blood loss. Our study indicated that hypofibrinogenemia of ≤150 mg/dL after protamine administration at the end of CPB may indicate the need for treatment to reduce postoperative blood loss.

The results of this study showed that the presence of cyanotic disease was associated with postoperative blood loss after adjusting for other potential confounders and variations in surgeon techniques. The presence of cyanotic disease has been recognized as a risk factor for postoperative coagulopathy and blood loss in the perioperative period of pediatric cardiac surgeries [6,12,13]. There are several potential explanations for this finding. First, patients with cyanotic heart disease may have thrombocytopenia as chronic hypoxemia stimulates erythropoiesis and the excess production of RBCs inhibits platelet production [14]. Second, deficiencies in platelet adhesion and aggregation may occur in patients with cyanosis [15]. Third, cyanosis may cause a deficiency of coagulation factors. Hypoxemia decreases the production of vitamin K-dependent coagulation factors [16]. Additionally, the sluggishness of local microcirculation due to high blood viscosity in cyanotic patients may cause coagulopathy [16]. Finally, cyanosis may decrease clot firmness in the fibrinogen/fibrin component of the clot. These factors may be associated with preoperative hypofibrinogenemia. A previous study showed that FIBTEM-MCF, the parameter indicating clot firmness in rotational thromboelastometry (ROTEM®︎; TEM International, Munich, Germany), was reduced in cyanotic pediatric patients who underwent cardiac surgeries [17,18]. Therefore, special care, including maintenance of fibrinogen level, may be needed for patients with cyanotic heart diseases to prevent major postoperative blood loss.

Surgeons’ technique was shown to be an independent risk factor for postoperative blood loss in adult cardiac surgeries [19]. Previous studies that showed the risk factors of postoperative blood loss in pediatric cardiac surgeries did not account for differences in surgical technique [2,3]. Moreover, the findings were not adjusted for potential confounders (e.g., age, presence of cyanotic disease, re-sternotomy, deep hypothermia during CPB, long CPB duration time, and operative complexity) [2,3]. Therefore, we applied a multilevel logistic regression model wherein surgeons’ technique was considered as a random effect, and potential confounders were adjusted to validate the risk factors of postoperative blood loss in pediatric cardiac surgeries. Our study showed that a fibrinogen concentration ≤150 mg/dL was associated with postoperative blood loss even after adjusting for the differences in the surgeons’ techniques and potential confounders.

This study has some important limitations owing to its retrospective nature. First, this study was conducted at a single institution. Additional multicenter prospective studies are warranted to improve generalizability. Second, unmeasured confounding factors may have influenced the findings. In particular, other coagulation factors associated with primary (e.g., von Willebrand factor) and secondary (factors II, V, VII, VIII, IX, and X) hemostasis were not adjusted for in this study. Third, the possibility of reporting bias cannot be eliminated as this study was based on data reported by anesthesiologists. Fourth, post-CPB transfusion did not follow a standard algorithm. Lastly, the validity of the definition of postoperative blood loss in this study was not sufficiently assessed in the previous studies. However, we applied the widely known definition of MBL-6 according to the NATA guidelines as a rational definition at present [11].

## Conclusions

This retrospective cohort study demonstrated that fibrinogen concentration ≤150 mg/dL after protamine administration at the end of CPB and the presence of cyanotic disease were associated with MBL-6. Maintenance of fibrinogen concentration >150 mg/dL after protamine administration at the end of CPB is recommended, especially for patients with cyanotic heart disease. Further prospective investigations are required to improve the validity of our study results.
